# Hospital governance accountability structure: a scoping review

**DOI:** 10.1186/s12913-023-10135-0

**Published:** 2024-01-10

**Authors:** Mohammad Ali Jalilvand, Ahmad Reza Raeisi, Nasrin Shaarbafchizadeh

**Affiliations:** 1 Student Research Committee, School of Management and Medical Information Sciences, Isfahan University of Medical Sciences, Isfahan, Iran; 2 Health Management and Economics Research Center, Isfahan University of Medical Sciences, Isfahan, Iran; 3 Health Services Management, Hospital Management Research Center, Health Management Research Institute, Iran University of Medical Sciences, Tehran, Iran

**Keywords:** Public hospital, Accountability, Governance, Structure

## Abstract

**Introduction:**

Hospitals, as complex organizations with clinical, financial, and social functions, face different barriers to providing high-quality and safe services at reasonable costs. Various initiatives have been carried out in hospital governance to improve quality, safety, and accountability. This research aims to identify the structures and dimensions that make hospital governance accountable.

**Methods:**

The research used Arksey and O'Malley's scoping review framework to examine the research literature on hospital governance structure and accountability. The literature review included PubMed, Web of Science, Embase, Scopus ProQuest, Google search engine, and Google Scholar databases from 2010 to 2023. Data were analyzed using the content analysis method.

**Results:**

Excluding unrelated and duplicate sources, 40 articles and reports were included in the study. The studies were reviewed and analyzed based on organizational type, type of source, year of publication, objectives, and key findings. Accountable governance features were extracted from the selected articles and reports. The four main themes include inclusive governance, commitment to accountability, planning for accountability, and autonomous governance. Thirteen subthemes were extracted from the study literature.

**Conclusion:**

Various initiatives have been implemented regarding the reform of the governance structure of public hospitals in different countries. Many of these reforms aim to improve financial and clinical accountability. The study results could be used to identify the structures and dimensions that make hospital governance accountable.

**Supplementary Information:**

The online version contains supplementary material available at 10.1186/s12913-023-10135-0.

## Introduction

One of the fundamental factors of an organization's success is accountability [1]. Accountability in organizations that provide health services is defined as the responsibility of an individual or an organization regarding its actions and performance [2]. Accountability in hospitals refers to responsibility for the overall quality and safety of care [3]. Hospital governance can be defined as the set of structures and processes that define the strategic direction for the hospital and the means by which resources are assembled and allocated to achieve them [4]. Hospital governing bodies have a fundamental role in overseeing quality and safety by defining priorities and objectives, crafting strategy, shaping culture, and designing organizational control systems [3]. An accountability regime will always be based on three elements: a clear definition of desirable goals or objectives (the object of accountability), the ability to measure and monitor goal achievement, and a set of consequences for providers or organizations if achievements regarding goals or objectives are not satisfactory [5] In recent years, improving the accountability of healthcare organizations has been one of the main motivations for reforms in health systems [2]. Studies have introduced accountability as a tool for increasing transparency and improving healthcare quality [6, 7].

However, in developing countries, health sector reforms have concentrated primarily on increasing financial accountability and have paid less attention to other forms of organizational accountability [8]. The Health Services Delivery Program of the World Health Organization introduces five components of accountability: a) legal accountability, which includes planning, contracting, and budgeting; b) financial accountability, which includes tracking and reporting on fund allocations, funds disbursement, and ethical use of resources; c) professional accountability, which promotes service delivery according to legal, ethical, and professional standards; d) political accountability, which ensures that governments fulfill public trust, represent the public's interest, and respond to societal needs and concerns; and e) public accountability, which includes public engagement at all levels and appropriate structures to support information flow between decision-makers and different public involvement fora [9].

The concept of accountability has traditionally been drawn somewhat narrowly by public lawyers to encompass the formal duties of public bodies to account for their actions to ministers, parliament, and courts [10], so a common understanding of hospital governance accountability is where an upstream entity such as a government, regional health agency, board of trustees, or professional association can hold providers or organizations accountable for achieving specific goals or objectives [5]. Hospital governance might promote or undermine health performance [11, 12]. The governance structure defines the strategic direction, objectives, policies, legislation, regulations, and programs and monitors and assesses their achievement [13]. In this study, we consider both internal and external accountability.

Hospital governance leadership differs from other institutions or industries [14–17]. The external environment of the hospital is constantly under the pressure of public opinion and governments to spend resources more efficiently [14]. The internal environment of the hospital has various independent specialists, such as physicians, nurses, paramedics, financial affairs, and management professionals, who communicate and make them accountable for achieving hospital goals, which is a complex matter [18].

Hospital leaders should question whether the current organizational governance structure is optimal for converting inputs into clinical and financial outputs [19–21]. The perceived problems in ensuring the accountability of hospitals and the efficiency of their performance led governments to various governance structure initiatives. Autonomous hospitals, corporate hospitals, hospitals with boards of trustees, hospitals with public‒private partnerships, and budgetary hospitals are examples of these structural initiatives [22, 23]. These structural reform initiatives had different positive and negative outcomes [23].

Clinical professionals try to achieve health goals, and the management team tries to achieve financial and management goals in their way. This shows the necessity for aligning these teams to a specific and robust hospital administration and management structure. The point where many of them are the most critical problem in hospitals is existing clinical and nonclinical parts and making both parts accountable [18, 24, 25]. Hospital governance refers to the balancing mechanisms and controls that shape the decision-making process in hospitals. Clinical participation and management professionalism are essential aspects of the governance structure in a hospital [20]. Clinical and nonclinical actors have different patterns of thinking and doing at different organizational levels in hospital districts and different perspectives on accountability. In this setting, the gap between managerial identity accountability (i.e., to comply with governance policies issued by the political institution) and the accountability of medical professionals in their domain leads to accountability tensions [26].

Hospital decision-making is a complex and often diffuse process involving key people, including physicians, administrators, and boards. Physicians are essential in clinical decisions and should be accountable for high-quality, safe care. Administrators influence hospital policy and planning activities [27]. Medical staff usually involved in the hospital governance structure as board members or organized in a separate structure have been called under various names, including medical staff council or medical advisory committee, to create accountable clinical departments [28, 29].

The most important task of the hospital is to provide safe and quality clinical services [30]. If it is not considered in the hospital's governing structure, the capacity for this function cannot be sure of its accountability [31]. Hospital governance can only be fully understood by taking the role of the medical staff into account. Therefore, hospitals should use medical staff as financial and planning committees in their governance structure [32]. As a result, while the administrative structures of the hospital are legally responsible for monitoring quality and safety, they delegate authority for monitoring quality and safety in the scope of the medical staff council [21].

A typical model of hospital governance to overcome accountability problems is adding upstream structures, such as the Board of Trustees, to the hospital governance structure [33, 34]. Having a professional, vigilant, independent board greatly impacts the performance of any organization, including hospitals [35]. Empowered by a strong and effective board, a well-performing hospital will be able to ensure that social obligations can be fulfilled and that patients will receive proper treatment and care while maintaining economic and financial sustainability [36]. Some studies show that a common factor in the inefficiency of various governance structures is weakness in monitoring and responding to quality issues in public hospitals [37]. The importance of governance for the accountability of health systems is broadly recognized. Despite this recognition, accountable governance definitions continue to be disputed, and arguments and confusion persist about how governance structure interventions influence hospitals' accountability and health outcomes. Governance-related linkages or interventions often need to be better understood and documented. This lack of evidence can result in reticence and hesitation to invest in hospital governance structure improvements or overreliance on a limited set of successful governance interventions [36, 38].  An accountable governance structure is necessary because public hospitals play an essential role in health systems. However, identifying the characteristics and activities of this structure to achieve accountability has received less attention. The present study aimed to identify accountable governance structures in public hospitals through a scoping review of the global research literature.

## Method

### Eligibility criteria

The present study uses the Arksey and O'Malley framework to examine the extent, range, and nature of research activity on the accountable governance structure in public hospitals. Arksey and O’Malley’s framework includes six stages, the sixth being optional: a) identifying the research question that it is necessary to include three parts of the research question: a) Population b) Concept c) Context (PCC question); b) identifying relevant studies, a process that is as comprehensive as possible; c) study selection, with the establishment of inclusion/exclusion criteria, based on familiarity with the literature; d) charting the data, a stage that includes sifting, charting, and sorting information according to key issues and themes; e) collating, summarizing, and reporting the results, which provides both a descriptive and numerical summary of the data and a thematic analysis; and f) a consultation exercise, an additional, parallel step involving key stakeholders to inform and validate study findings [39]. Peer-reviewed papers and the gray literature (government reports, policy documents, reports of consultants, unpublished reports) were written in English between 2010 and January 2023. Databases of ongoing research and unpublished literature were searched. According to the research question, studies about P= public hospitals, C=accountable governance, and C= all over the world were included in the study. Other inclusion criteria were published after 2010 and written in English. For gray literature, the inclusion criteria were free online full-text versions, English language, documents related to public hospitals, and the study's time frame. The exclusion criteria were missing full text, articles focused on nonhospital entities, and papers focused on accountable care organizations.

### Information sources

The PubMed, Web Of Science, Embase, Scopus ProQuest databases, and Google Scholar search engine were searched. The Google search engine obtains gray literature, including reports, regulations, guidelines, and policies. Google search without a time limit based on the first 200 results added to the search regardless of date. To determine the keywords of the research, after searching the sources and consulting with the experts, the three main concepts of accountability, governance, hospital, and their synonyms were searched in different databases. The search strategies in different databases are reviewed based on the characteristics of the database and presented in Table 1.

**Table 1:** Search strategy summary for the scoping review

Google	((Accountable OR accountability OR answerability OR liability OR account-giving) AND (governance* OR management OR plan OR administration OR leader OR organization OR structure)) AND (Hospital OR clinic OR “health centre” OR “health center”)	
Google Scholar	((Accountable OR accountability OR answerability OR liability OR account-giving) AND (govern* OR management OR plan* OR administration OR leader* OR oraganiz* OR structure*)) AND (Hospital* OR clinic* OR “health centre” OR “health center”)	946
Scopus	( TITLE ( ( accountable OR accountability OR answerability OR liability OR account-giving ) AND ( govern* OR management OR plan* OR administration OR leader* OR oraganiz* OR structure* ) ) AND TITLE-ABS-KEY ( hospital* OR clinic* OR "health center" OR "health center" ) )	127
Proquest	ti((Accountable OR accountability OR answerability OR liability OR account-giving) AND (govern* OR management OR plan* OR administration OR leader* OR oraganiz* OR structure*)) AND (Hospital* OR clinic* OR "health centre" OR "health center")	56
Web of Science	(Accountable OR accountability OR answerability OR liability OR account-giving) AND (govern* OR management OR plan* OR administration OR leader* OR oraganiz* OR structure*) AND (Title) and Hospital* OR clinic* OR “health centre” OR “health center” (All Fields)	82
PubMed	(Accountable[Title] OR accountability[Title] OR answerability[Title] OR liability[Title] OR account-giving[Title]) AND (govern*[Title] OR management[Title] OR plan*[Title] OR administration[Title] OR leader*[Title] OR oraganiz*[Title] OR structure*[Title])	232
Embase	(accountable:ti OR accountability:ti OR answerability:ti OR liability:ti OR 'account giving':ti) AND (govern*:ti OR management:ti OR plan*:ti OR administration:ti OR leader*:ti OR oraganiz*:ti OR structure*:ti) AND (hospital*:ti,ab,kw OR clinic*:ti,ab,kw OR 'health centre':ti,ab,kw OR 'health center':ti,ab,kw) AND (2010:py OR 2011:py OR 2012:py OR 2013:py OR 2014:py OR 2015:py OR 2016:py OR 2017:py OR 2018:py OR 2019:py OR 2020:py OR 2021:py OR 2022:py)	85

### Selection of sources of evidence

**Fig. 1:**
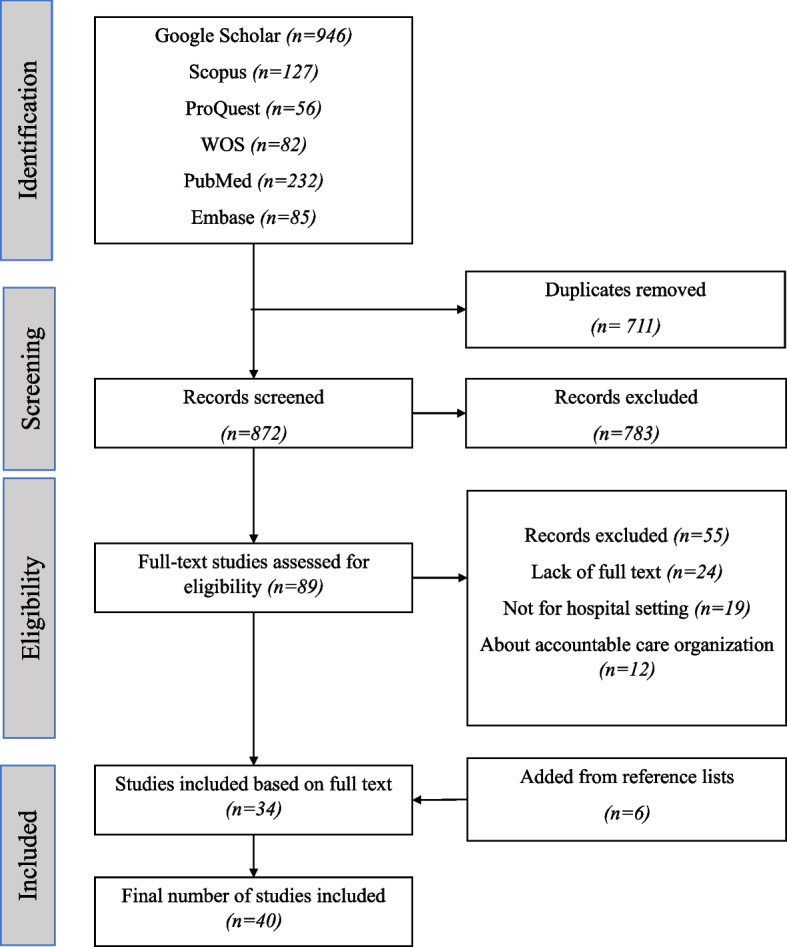
PRISMA flow diagram for included studies

First, we entered the literature search from different databases into Mendeley software. The remaining sources are examined after removing the duplicates. Titles and abstracts were screened. In this stage, articles were excluded if they were not relevant. In the next stage, the full text of the remaining sources is screened. Moreover, they were excluded from the study if there were no relevance or exclusion criteria. Finally, the remaining articles were reviewed (Fig 1).

The included sources were reviewed. A summary was prepared. Key points of each reference were identified, and a summary, including the first author's name, the date of publication, the country, the purpose, and the main findings, was prepared (Table 2).

**Table 2:** Characteristics of the included studies in the scoping review in the order of publication date

**Authors, country, year**	**Objective/Focus**	setting	**Document type/Methodology**	**Key points**
Goeschel [48] USA 2010	review these responsibilities, describe opportunities for boards and medical staffs to collaborate as leaders, and offer recommendations for how boards and medical staff members can address the challenges of shared responsibility for quality of care.	US hospitals	Peer review article/ literature review	Boards need to hold CEOs and medical staff leaders accountable for improvements on both kinds of measures and ensure that the institution has the resources and will to improve
Dixon [52] England 2010	To map and describe the formal accountability relationships of foundation trusts in England	Six acute trusts	Peer review article/qualitative	Directors of foundation trusts perceive strong accountable to their regulator, Monitor, particularly for financial performance, but there is some confusion about where accountability for quality of care rests. Horizontal lines of accountability to the local population (through Local Involvement Networks and local government Overview and Scrutiny Committees) remain weak.
Connor [81] world 2011	define and describe accountability as a key component of clinical governance and a responsive, fair and transparent health culture.	Healthcare systems	Peer review article/literature review	such resolution enables the individual and organization to learn and patients, families and communities to continue to trust in the healthcare system
Jiang [53] United States 2012	provide an update to prior research by exploring the role and practices of governing boards in quality oversight through the lens of agency theory and comparing hospital quality performance in relation to the adoption of those practices	445 public and private not-for-profit hospitals	Peer review article/quantitative	Hospital governing boards should examine their current practices and consider adopting those that would enhance the accountability of the Board itself, management, and the medical staff.
Ahlin [1] USA 2012	establishes the broad vision and goals of the organization.	Ontario Hospital Association	Report	The Board of Directors must include the CEO, the President of the medical staff, the Chair of the Medical Advisory Committee and the Chief Nursing Executive.
World Health Organization [60] world 2012	Identify innovations for healthcare governance in 21 centuries	Health system	report	Healthcare governance should be accountable and health governance should contain community representee
Mattei [46] Germany, Norway and Denmark 2013	the impact of hospital reforms on accountability relations in three health systems by focusing on investment decisions within healthcare. The link between accountability form	public hospitals	Peer review article/literature review	National governments have tightened their control over the overall trajectory of their hospital systems, but have also shifted significant responsibility downward to the hospital level
Kaini [59] Nepal 2013	focus on regulation and accountability aspects of the healthcare governance agenda	Nepal healthcare	Peer review article/qualitative	the concept of healthcare governance has to be formally introduced by the government and other health authorities including professional bodies and councils in Nepal by introducing healthcare governance strategies and policies.
Jha [54] USA England 2013	How do the governance practices among the boards overseeing English hospitals differ from those of boards of directors of US hospitals? How does a trust's having foundation status affect governance practices in English hospitals? And third, are the associations between engaged governance and higher quality performance that are seen in US hospitals also apparent in England?	171 board chairs in public hospital	Peer review article/quantitative	there is room for improvement in both countries to bolster board expertise and focus on key quality metrics, and to hold managers accountable for the delivery of safe, effective health care
Zenty [50] USA 2014	anticipate the dramatic changes of healthcare reform	University Hospitals Health System	Peer review article/review	thoughtful governance, effective plan design, customized data analytics, physician networks and incentives, innovative patient engagement, and supplemental coordination resources
*Vaughn* [51] *USA 2014*	Do governing boards, C-suites, and clinical management possess different perceptions regarding structures, processes, and quality related activities in their hospital?	300 hospitals were linked to performance on the Centers for Medicare & Medicaid Services (CMS)	Peer review article/quantitative	major organizational drivers of quality and safety: (1) commitment of senior leaders, (2) a vision of exemplary quality, (3) a supportive culture, (4) accountable leadership, (5) appropriate organizational structures, and (6) adaptive capability.
MacDonald [49] England 2014	explore the experience of service user governors in foundation trusts and their capacity to hold boards to account	National Health Foundation Trusts	Peer review article/qualitative	emerged concerned: the role of a governor, conduct and content of meetings, agenda setting, relationships and representation.
Government of the Northwest Territories [67] Canada, 2014	overall accountability framework that outlines performance reporting requirements for key target audiences, including timing, indicators, and data collection responsibilities, and creation of an action plan to implement the accountability framework and performance measurement system.	Health and Social Service Authorities	report	Guiding the development of the performance indicators was the understanding that they were meant to be at the “system” level rather than the HSSA level
The Healthcare Governance & Transparency Association Egypt 2014 [36] Egypt 2014	Guide offers Principles and Guidelines to facilitate the incorporation of corporate governance practices in hospitals in Egypt	hospitals in Egypt	report	This Guide supports individual hospitals in responsibly and sustainably increasing their performance through the incorporation of corporate governance practices
Szekendi [58] USA 2015	governance structures and practices, influence health care quality.	US academic medical centers	Peer review article/qualitative	All hospitals, even those with the highest quality ratings, had major gaps in their use of best practices for CEO and board assessments. the relationship between use of these practices and organizational performance, based on the University HealthSystem Consortium’s Quality & Accountability rankings
Pascal [82]France 2015	History of Hospital Reform and Accountability in France	Hospitals in France	Peer review article/narrative review	reconciliation between economic and medical requires the establishment of “mediconomic” tools permitting the evaluation, in a language understood by all, by means of quantitative and qualitative medical and economic indicators built from collective understanding of workplace realities
Nyland [45] Norway 2015	why public sector reforms hybridize during implementation processes, consequences on accountability relations and practitioners’ and policymakers’ reactions to these changes	Norwegian hospital sector	Peer review article/case study	the gradually alignment of controls in a dynamic pattern of hybridization enables the balancing of conflicts in the chain of accountabilities. Hybrid controls are observed to emerge as stronger than the initial ideal control models
Mutigand [26] Finland 2015	Analyses the impact of the institutionalization of governance and budgetary Policies on the accountability of organizational actors	Two public hospitals	Peer review article/critical realism	The political and the technical. Accountability practices depend on how the institutionalized policies have reduced or increased the gaps between the real, the actual and the empirical domains of reality of the organizational actors involved and the governance policy that prevails at a given domain of reality
Askim [69] Norway 2015	How are administrative and managerial accountability combined, and to what extent does it depend on agency characteristics?	five state agencies in Norway in the area of hospital administration, welfare administration, and immigration.	Peer review article/case study	more insight into the differences between administrative and managerial accountability and the interplay between them.
Jones [56] England 2016	how boards govern for quality improvement	15 healthcare provider organizations	Peer review article/qualitative	boards with higher levels of maturity in relation to governing for QI had the following characteristics: explicitly prioritizing QI; balancing short-term (external) priorities with long-term (internal) investment in QI; using data for QI, not just quality assurance; engaging staff and patients in QI; and encouraging a culture of continuous improvement.
George [64] Nigeria 2016	develop a framework that highlights mutually reinforcing dimensions of accountability in health systems along three counterbalancing axes	Nigeria primarily serves	Peer review article/qualitative	Reframing accountability as a means of sparking, supporting and steering change can highlight different dimensions of health systems that need reform, particularly depending on the positionality of the viewpoints consulted.
Health Service Executive Ireland 2016 [66]	performance accountability framework for the health services	Dr. Steeven’s Hospital	report	introduce Accountability levels, Accountability Suite (Plans, Agreements and Reports) , Accountability processes, Escalation and Intervention Framework 2016
Rosen [83]USA 2017	Creating a Pediatric Joint Council to Promote Patient Safety and Quality,	Johns Hopkins Medicine	Peer review article/case study	a focused structure for coordinated efforts across disparate pediatric entities, ensuring horizontal peer learning and entity-specific improvements, as well as vertical lines of accountability and central oversight with shared governance
Pronovost [42] USA 2017	offer six principles that health system leaders can apply to establish a governance and management system for the quality of care and patient safety.	Johns Hopkins Medicine	Peer review article/qualitative	ensure there is oversight for quality everywhere care is delivered under the health system; create a framework to organize and report the work; identify care areas where quality is ambiguous or underdeveloped (i.e., islands of quality) and work to ensure there is reporting and accountability for quality measures; create a consolidated quality statement similar to a financial statement; ensure the integrity of the data used to measure and report quality and safety performance; and transparently report performance and create an explicit accountability model
Mannion [84] England 2017	validate the structure of an established ‘Board competencies’ self-assessment instrument in the English NHS relationships between (a) Board competencies and staff perceptions about how well their organization deals with quality and safety issues; Board competencies and a raft of patient safety and quality measures at organization level	95 acute hospitals	Peer review article/quantitative	better Board competencies were correlated in consistent ways with beneficial staff attitudes to the reporting and handling of quality and safety issues (using routinely collected data from the NHS National Staff Survey).
Geyndt [68] Iran Tunisia Zambia Dominican Republic Uganda Ecuador Indonesia Malaysia Kenya 2017	(a) synthesize the experience of eleven countries at granting autonomy to their public hospitals and the obstacles encountered; (b) deduce which autonomy policies have or have not been effective documenting successes and failures; and (c) propose evidence-based recommendations to policy makers.	Public autonomous hospital	Peer review article/comparative	Governance of autonomized hospitals by Boards however is obstructed by the resistance of central level entities to have their authority diminished. The Ministry of Finance tends to maintain control over revenues and expenditures. Decentralizing decision making to the operational level has had limited success.
Austin [43] United States 2017	How the Application of Financial Structures to Safety and Quality Can Drive Accountability in a Large Health Care System	John Hopkins Medicine	Peer review article/literature review	The four components implemented by Johns Hopkins Medicine were governance, accountability, reporting of consolidated quality performance statements, and auditing.
Reich [71] USA 2018	introduces a simplified model for assessing and designing the governance of global health public–private partnerships	PPP in global health	Peer review article/review the literature	The matrix is proposed to improve conceptual clarity and help identify concrete options for action in planning, assessing, and adjusting PPP governance.
Rechel [62] EU 2018	the brief explores: • ownership and legal form of hospitals (private or public, organized as a trust, for-profit or not-for-profit, etc.) • strategic planning of hospital infrastructure and capital investment at the national, regional or subregional government level • degree of decentralization of hospital governance (hospital governance layers between the Ministry of Health and the hospitals; political representation versus administrative responsibility; extent of direct managerial control by higher administrative structures).	10 European countries: Denmark, England, Finland, France, Germany, Italy, Netherlands, Scotland, Spain and Sweden.	Policy Brief/rapid review	here are two basic types of decentralized system. Many countries in Europe already have decentralized health systems where hospital governance is the responsibility of subnational bodies. This is the result of long-standing historical processes rather than explicit policy-making. In other countries, health systems have been actively decentralized, either as part of wider political changes or as part of a specific package of reforms.
Botje [19] Netherlands 2013	describe hospital governance and the quality orientation in the Netherlands. Also we wished to investigate the relationship with hospital performance.	Dutch hospitals	Peer review article/cross-sectional	boards of trustees and management boards had a reasonable quality orientation. Boards were familiar with quality guidelines, received a reasonable amount of information related to quality and used this for monitoring quality and policy-making.
Bonde [85] Denmark 2018	analyses an experiment into healthcare governance in Denmark inspired by principles of value-based healthcare and intended to reorient the focus of healthcare governance from 'productivity' to 'value for the patient'	9 hospital departments	Peer review article/experimental study	the locally developed indicators facilitated what we call 'dialogical accountability', and we discuss whether this represents a feasible way forward for value-based health care.
Kuhlmanna [55] Russia 2019	the role of physicians within the managerial structure of Russian hospitals.	19 public hospitals	Peer review article/qualitative	three major problems of hospital management in the Russian Fed-eration. First, hospitals exhibit a leaky system of coordination with a lack of structures for horizontal exchange of information within the hospitals (meso-level). Second, at the macrolevel, the governance system includes implementation gaps, lacking mechanisms for coordination between hospitals that may reinforce existing inequalities in service provision. Third, there is little evidence of a learning culture, and consequently, a risk that the same mistakes could be made repeatedly
Glenngrd [47] Sweden 2019	contribute to knowledge about what is regarded as an appropriate governance model in welfare markets in healthcare, from the perspective of government.	Swedish primary care	Peer review article/literature review	Using management controls in a way that improves the providers’ attitude toward and capacity to achieve the assigned task of delivering high-quality healthcare was described as central
Vian [61] world 2020	summarize concepts, frameworks, and approaches used to identify corruption risks and consequences of corruption on health systems and outcomes.	critical review based on a systematic search of literature	Peer review article/critical review	show how anti-corruption strategies such as transparency, accountability, and civic participation can affect corruption risk. Ghost workers and absenteeism Dual practice and corruption risks
Uddin [44] Japan 2020	examine healthcare governance and its implementation in the historical, institutional and cultural context of Japan	Japanese public hospital	Peer review article/data triangulation conducting documentary research, observations and semistructured interviews	The role of healthcare professionals is crucial in execution of NPM reform in Japan - Western centric governance reform departed significantly from its idealized form - The power of medical school and the ikyoku shape the governance process - Corporate Board maintains harmony instead of seeking accountability
AlMubarak, [80] KSA 2020	explore the perceptions of different stakeholders about the privatization of the Saudi health care system.	a public hospital	Peer review article/qualitative case study	The first was pertinent to the changes in the governance structure, with gradually increased autonomy from the government. The second reflected the necessity to introduce accountability within hospitals. The third described the cooperative relationship among the E1-Cluster hospitals as well as its competitive relationship with the private sector
Local Health Integration Network [65] Canada 2020	contractual performance targets	Middlesex Hospital Alliance	Report	stipulates accountability and performance obligations for planning, integration and delivery of programs and services.
Alhothaly [86] KSA 2021	identify the accountability in health care organization from patients	King Abdullah Medical City (KAMC)	Peer review article/Cross-Sectional Design	there is significant relationship between accountability in healthcare and dimensions such as professionals in healthcare, Government actions, legal and ethical concerns, and administration and management actions. The correlation matrix and regression analysis show that all the four dimensions have strong correlation with accountability in healthcare settings
Fayed [57] Egypt 2021	compare the governance structures and practices in for profit and nonnon-for-profit hospitals in Alexandria, Egypt.	For-profit and nonnon-for profit hospitals	Peer review article/e/ descriptive cross-sectional study	As for private hospitals, Board existed in only 72 hospitals (82.75%5 %). Almost all boards have CEO duality. Board members were as few as two members in some boards and up to twenty members in others. Some hospital boards did not have an orientation manual or program.
Kesale [63] Tanzania 2022	analyze the status of accountability of Health Facility Governing Committees in Tanzania under the Direct Health Facility Financing setting as perceived by the supply side.	32 different health institutions	Peer review article/qualitative and quantitative	The health facility governance committee’s responsibility was shown to be substantially connected with the health planning component (*p* = 0.0048) and the financial management aspect (*p* = 0.0045).

### Critical appraisal of individual sources of evidence

A scoping study will need some analytic framework or thematic construction to present a narrative account of the literature. There is no attempt to present a view regarding the 'weight' of evidence about particular interventions or policies [39]. Per guidance on conducting scoping reviews and consistent with scoping reviews on health-related topics, the methodological quality of the included reports was not appraised [39, 40].

### Synthesis of results

The studies were categorized based on the characteristics of the hospital governance structure, methodology, settings, and key findings. Then, topic construction was performed using the attributes of various hospital governance structures (Table 2). The strategy of data analysis in the present study is qualitative content analysis. Using qualitative content analysis is one of the usual methods for synthesizing results in scoping review studies. This method helps to obtain a summary of the data by coding [41]. The research identifies the components of governance structure and accountability of hospitals worldwide.

## Results

Of the 40 sources included in the study, 85% were published in peer-reviewed journals. Moreover, 15% had organizational reports at the national or international level. Sixty-two percent of the sources were published between 2015 and 2022. Seventy-five percent were about the governance and accountability structure in hospitals, and 25% were about hospitals and other health system components. A total of 27.5% of the studies were conducted in the United States, and 37.5% were conducted in European countries and the United Kingdom. A total of 22.5% of the studies were completed with quantitative research methods and the same amount with qualitative research methods. Table 3 describes the sources used (Table 3).

**Table 3:** Description of included studies

Variables	N (%)
Publication Type	
Journal article	33(82.5%)
Report	7(17.5%)
Publication date	
2010-2014	15(37.5%)
2015-2022	25(62.5)
Setting	
Hospital	30(75%)
Healthcare system	10(25%)
Country:	
USA	11(27.5%)
UK	5(12.5%)
EU countries	10(25%)
world	3(7.5%)
other	11(27.5%)
Methodology:	
Qualitative	10(25%)
Quantitative	7(17.5%)
Review	6(15%)
Report	6(15%)
Case Study	5(12.5%)
Critical Realism	2(5%)
Mixed-Method	1(2.5%)
Comprehensive Review	1(2.5%)
Rapid review	1(2.5%)
Experimental Study	1(2.5%)

After categorizing and analyzing the data, four main themes and thirteen subthemes were extracted (Table 4).

**Table 4:** The main themes and subthemes of the study

Main themes	Subthemes
inclusive governance	Hospital board (of trustee, governance, or director) [1, 42–52]
	Committees [26, 46, 52–54]
	Medical staff [1, 26, 44, 45, 48, 50, 51, 55–57]
	Nurse representative [50, 51, 54, 58]
	Community representative [47, 49, 50, 55, 59, 60]
Commitment to accountability	clinical accountability [52, 61, 62]
	Financial accountability [44–46, 48, 52, 61, 63]
	Social and Political accountability [45, 46, 52, 61, 64]
Planning for accountability	Accountability plan [43, 48, 58, 65–67]
	Clear report line [44, 47, 50, 52, 55, 67]
Independent governance	Decentralized [26, 46, 52]
	Autonomous [36, 52, 57, 68, 69]
	Hybrid governance [47, 52, 54, 55, 69]

### Inclusive governance

Hospitals are complex entities. Public hospitals face pressure from public opinion and politicians to be accountable for public resource usage. Additionally, the internal environment of the hospital faces various specialties. Management, economics experts, and medical and nursing staff try to achieve their goals. Aligning financial and clinical goals is challenging for hospital governance [26]. In addition, the clinical and administrative departments probably cannot be accountable to each other, so an intermediate structure is necessary to make the two departments accountable simultaneously [18, 24, 26]. For this purpose, the public hospital should use the presence of a board in its structure [19, 43, 53]. This Board can be called the Board of Trustees, the Governing Board, or the Board of Directors [42–48]. This structure can have medical staff members or a counterpart structure called the council of medical staff [26, 44, 45, 48, 50, 51, 55–57]. f [49, 50, 55, 59, 60]. Additionally, the presence of nurses' representatives in the governance structure helps to make nursing and paramedical departments accountable [50, 51, 54, 58]. Forming various hospital committees also helps minimize the gap between clinical and nonclinical groups. These committees lead to a common understanding of barriers and resources [26, 46, 52–54].

### Commitment to accountability

Accountability has different dimensions and forms. However, developing countries are primarily satisfied with defining it as a financial issue [70]. Public hospitals consume public funds, so the public population is defined as its stakeholders [8, 44–46, 48, 52, 60, 61, 63]. This definition ignores their role in providing health care as the essential function of hospitals. Therefore, it needs to be considered a comprehensive definition. An essential part of hospitals' accountability is their clinical accountability. The hospital's governance structure should be committed to providing safe and high-quality care and evaluating its achievement [52, 61, 71]. Another type of accountability is the political and social accountability of the hospital. As a healthcare organization, the hospital has the role of political and social accountability and cannot ignore it. Social and political accountability includes the hospital's responsibility regarding social and political issues, emphasizing their most critical role, namely, the clinical role [45, 46, 52, 61, 64]. Social and political accountability refers to the degree to which governments and institutions deliver on promises, act in the best interest of citizens, and respond effectively to societal needs [61].

### Autonomy

In traditional accountability models, hospitals should be accountable to an upstream entity such as the Ministry of Health, local health department, or university. However, implementing external accountability is complex and may not be accurate [72]. In contrast, the hospital may have a structure that goes beyond the administrative bureaucracies of government organizations to monitor its performance and take necessary measures [52, 57, 68, 69]. The hospital should be able to be accountable to its governance structure (board, council of medical staff) for all performances, including financial, clinical, sociopolitical, strategies, and operations [26, 36, 46, 52]. Having a completely independent or hybrid governance structure is a way that hospitals follow to improve their accountability. The meaning of hybrid governance is that, in addition to having an internal structure for accountability, the hospital can also be accountable to the government parts, such as the Ministry of Health, for providing safe, quality services at a reasonable cost [47, 52, 54, 55, 69].

### Planning for accountability

The existence of a specific program for accountability helps all stakeholders in hospitals, including clinical and nonclinical staff, patients, and the community, to know their responsibilities and authorities. This document contains an articulated set of responsibilities and associated financial, clinical, social, and political accountabilities. Hospital staff, patients, and society know which kind of accountability structure is run in the hospital and how they can use it if needed [43, 48, 58, 65, 66]. The existence of a clear reporting line that allows each employee to know what structure and people they are responsible for helps to reduce confusion and allows employees to make correct decisions in sensitive situations and benefit from the advice of others. The reporting line will reach the hospital's governance structure, i.e., the board or council of medical staff. Employees must account for these structures regarding their performance [44, 47, 50, 52, 55, 66, 67].

## Discussion

Accountability is an essential part of social relations in societies. Individuals and organizations must be held accountable for what they do or do not do. Organizations that use public funds require high levels of accountability and transparency. Accountability ensures that public funds are properly allocated and closer to their predetermined results. The concept of accountability is considered a key concept in the health system. Since hospitals use the majority of health resources in all health systems, it is necessary to be accountable. The relationship between governance structure in the hospital and accountability is clear. The governance structure in public hospitals is required to provide a basis for achieving accountability in various areas.

The hospital's governance structure must be a combination of its key stakeholders. The presence of management and economic experts, doctors and nurses, and representatives from the community in the hospital's governance structure can effectively increase its accountability. These individuals can be present on the Board, its committees, or similar structures. Atuesta et al. have shown that hospitals whose governance structure also has medical groups can provide better quality and safer services [73]. Nurses are one of the most critical groups in hospitals. The present study emphasizes their role in the hospital governance structure. This finding is in accordance with the study of Esfandnia in Iran. This study also shows that the presence of nurses in the hospital governance structure can improve patient safety [74]. The presence of community representatives on the hospital board was considered in the present study. Wright also criticizes the structure of the hospital's Board of Trustees in Britain and calls for the role of community representatives to play earnest roles [75]. The presence of these categories can provide the report line to the specialized forces in each field. The hospital is a specialized structure, and the existence of different groups in it will probably require a governance structure that can professionally manage each department and person to become accountable for their performance regardless of the type of expertise.

Although there is no unique definition of accountability, different types have been identified in different studies. The commitment of the health system and hospital managers to financial, clinical, and social-political accountability is one of the important findings of this study. Commitment to accountability is an important issue for the hospital's primary goal: clinical accountability. These findings have been confirmed in Cornock's study. In that study, the vital role of the clinical accountability of health professionals was mentioned [76]. However, some studies, such as Cornelese's study, argue that if this account is in the form of accountability to other physicians and specialists because of the psychological influence of peers, it will have adverse effects when an error occurs [77]. In the present study, social-political accountability is a form of accountability considered necessary for hospitals. These findings in Gorji's study are also confirmed, emphasizing that even the hospital's clinical performance faces challenges without fulfilling its social and political responsibility [78]. However, studies such as Byrkjeflot recommend that this type of accountability should not limit the accountability of hospitals regarding their clinical performance [79]. Financial accountability improves the hospital's financial status and reduces its costs, and clinical accountability provides high-quality and safe services for consumers. Social and political accountability, in addition to facilitating the hospital's achievement of its goals, can play an essential role in providing political support. and social responsibility of the hospital. When the managers' commitment to accountability is considered as a whole, it can be hoped that the hospital can achieve organizational success and provide sufficient benefits to all stakeholders.

Hospitals are an important part of the health system. If it is a public hospital, it operates as a department under the supervision of the Ministry of Health or regional health organizations. This creates an opportunity for external accountability in the hospital. External accountability can be applied in all financial, clinical, and sociopolitical contexts. However, it probably will not be enough on its own. Public hospitals have a complex internal environment; this environment may cause external organizations to not be able to respond accurately. Therefore, attention should also be paid to internal accountability. Internal accountability requires some authority in the governance structure and makes the hospital's independence necessary in many financial and administrative matters. Many initiatives worldwide have been carried out to reduce hospitals' dependence on the government. Preker divides hospitals into five categories: budgetary, independent, corporate, nonprofit, and private [22]. Similar to the present study, Badr's research also emphasizes that it is necessary to have independence in the hospital's governance structure [80]. The hospital's independence can increase its internal accountability and the governance's ability to exercise authority. By exercising the power of governance, the hospital can take necessary actions in case of deviation from proper performance in the financial, clinical, and social-political fields according to specific regulations.

The accountability plan for the hospital, in which the responsibilities and duties of each person and department are clearly mentioned, prevents confusion in accountability and possible neglect of part of it. Clinicians, doctors and nurses, and society and political officials should clearly understand how the hospital responds and effectively benefits from the hospital's response. Austin argues that having an accountability program and transparent reporting structure will help make hospitals more accountable [43]. Different hospitals need different programs to respond in all dimensions, and these programs cannot be communicated in a single form by a high-ranking institution such as the Ministry of Health. In addition to holding different departments accountable, this program can also help the hospital in terms of execution because the limits of each person's duties and powers are clear in it, and the hospital's governance structure can ask the person or department about the performed functions and ask for a specific answer.

## Conclusion

Vast social and economic changes have made health systems inevitably face accountability challenges worldwide. Hospitals are one of the most important entities in the health system and can only continue their practical life with accountability. The accountability of the hospital is primarily related to its governance structure. The current research findings emphasize the four dimensions of responsive governance in the health system. First, the governance structure should be comprehensive and include management, medical, nursing, and community representatives. Second, the governance structure should be open to all types of accountability, including financial, clinical, and social-political accountability. Third, the hospital governance structure should be independent to exercise its sovereignty and power if needed while monitoring accountability. The fourth important issue will be the definition of an accountability plan for the hospital, in which the duties and authority of each department and each person are clear. It is suggested to research the quantitative determination of the impact of each of the themes of the present study on accountability and, ultimately, the quality of health services. Be made. Using the findings of the current research can help hospitals provide their services more responsively and achieve better health outcomes.

### Supplementary Information

**Additional file 1.**

## Data Availability

The Excel dataset is available in the supplementary materials; for further details, contact the corresponding author (A.R.).
